# Genotyping and Molecular Characterization of Hepatitis B Virus from Human Immunodeficiency Virus-Infected Individuals in Southern Africa

**DOI:** 10.1371/journal.pone.0046345

**Published:** 2012-09-28

**Authors:** Euphodia Makondo, Trevor G. Bell, Anna Kramvis

**Affiliations:** Hepatitis Virus Diversity Research Programme, Department of Internal Medicine, University of the Witwatersrand, Johannesburg, South Africa; University of Pretoria/NHLS TAD, South Africa

## Abstract

Hepatitis B virus (HBV) and human immunodeficiency virus (HIV) are hyperendemic in sub-Saharan Africa. The HBV genotypes prevailing in HIV-infected Africans are unknown. Our aim was to determine the HBV genotypes in HIV-infected participants and to identify clinically significant HBV mutations. From 71 HBV DNA^+ve^ HIV-infected participants, 49 basic core promoter/precore (BCP/PC) and 29 complete S regions were successfully sequenced. Following phylogenetic analysis of 29 specimens in the complete S region, 28 belonged to subgenotype A1 and one to D3. Mutations affecting HBeAg expression at the transcriptional (1762T1764A), translational (Kozak 1809–1812, initiation 1814–1816, G1896A with C1858T), or post translational levels (G1862T), were responsible for the high HBeAg-negativity observed. The G1862T mutation occurred only in subgenotype A1 isolates, which were found in one third (7/21) of HBsAg^−ve^ participants, but in none of the 18 HBsAg^+ve^ participants (p<0.05). Pre-S deletion mutants were detected in four HBsAg^+ve^ and one HBsAg^−ve^ participant/s. The following mutations occurred significantly more frequently in HBV isolated in this study than in strains of the same cluster of the phylogenetic tree: ps1F25L, ps1V88L/A; ps2Q10R, ps2 R48K/T, ps2A53V and sQ129R/H, sQ164A/V/G/D, sV168A and sS174N (p<0.05). ps1I48V/T occurred more frequently in females than males (p<0.05). Isolates with sV168A occurred more frequently in participants with viral loads >200 IU per ml (p<0.05) and only sS174N occurred more frequently in HBsAg^−ve^ than in HBsAg^+ve^ individuals (p<0.05). Prior to initiation of ART, ten percent, 3 of 29 isolates sequenced, had drug resistance mutations rtV173L, rtL180M+rtM204V and rtV214A, respectively. This study has provided important information on the molecular characteristics of HBV in HIV-infected southern Africans prior to ART initiation, which has important clinical relevance in the management of HBV/HIV co-infection in our unique setting.

## Introduction

Hepatitis B virus (HBV), with a genome of ∼3,200 base pairs, is the smallest DNA virus infecting humans, yet it is one of the most important human pathogens, causing major health problems globally. HBV, the prototype member of the family *Hepadnaviridae,* is endemic in several parts of the world, including sub-Saharan Africa, which accounts for at least 65 of the 360 million people in the world chronically infected with the virus [1]. HBV causes chronic and acute infections, associated with severe liver diseases, including hepatitis, hepatic fibrosis, cirrhosis, and hepatocellular carcinoma (HCC). Moreover, of the 33.3 million adults and children living with HIV globally, 22.5 million reside in sub-Saharan Africa [2]. HIV infection leads to the acquired immunodeficiency syndrome [3], opportunistic infections, and premature death.

The two viruses share a common mode of transmission and can co-exist in the same host [4], and thus HBV and HIV co-infections are frequent in sub-Saharan Africa [5]. Because HBV infection precedes HIV infection in sub-Saharan Africa [5], the HBV exposure rate does not differ from that found in HBV mono-infected [6]–[9]. Even though direct comparison between studies is difficult because of differences in study design and geographical regions, a range of 28% to 99.8% exposure to HBV and 0.4% to 23% HBsAg prevalence have been found in HIV-infected South African cohorts [10]–[20]. Moreover, comparisons between HBV mono-infected and HBV/HIV co-infected individuals are further confounded by the fact that since the introduction of universal HBV vaccination in April 1995, no comprehensive studies have been carried out in South Africa to determine either the exposure or prevalence rates of HBV infection.

We have recently shown that of approximately 300 HIV-infected individuals from a rural cohort in Mpumalanga Province, 77.5% had at least one HBV marker, with 53.7% being HBVDNA^−ve^ (having resolved the infection) and 23.8% being HBVDNA^+ve^ [20]. HBV DNA without HBsAg, was detected in 15.1% of the participants [20], which is within the 8% to 18% range for other South African HIV^+ve^ cohorts [11]–[14], [16]. However, only three of these HBsAg^−ve^ participants met the “Taormina” definition of true occult HBV infection (HBV viral load <200 IU/ml) [21], whereas the remaining were *HBsAg-covert* (HBsAg-***c***ryptic ***o***vert) infections, having higher viral loads (>200 IU/ml) [20].

HBV replicates by reverse transcription of the pregenomic RNA using a viral-encoded polymerase that lacks proof-reading activity. The genome may evolve at an estimated error rate of 1.4–5×10^−5^ nucleotide substitutions/site/year, which results in genetic heterogeneity [22]–[24]. As a result of this genetic variability, genotypes of HBV have been identified defined by inter-genotypic differences of more than 7.5% in the complete nucleotide sequence [25]–[27]. To date, phylogenetic analysis of the HBV genome has lead to recognition of nine genotypes of HBV: A to D [26], [28], genotype E to F [25], [28], [29], genotype G [30], genotype H [31] genotype I [32]–[36]; and a tenth genotype J has been proposed [37]. The genotypes have distinct geographical distributions [38]. In Africa, genotype A is found predominantly in southern, eastern and central Africa, genotype D in northern Africa, whereas the majority of isolates from western Africa belong to genotype E [1]. Subgenotypes have been identified within genotypes A and D [26]. Genotypes A and D coexist in southern Africa, with genotype A predominating, with the dominant subgenotypes being A1 and D3 [39].

Very few studies have been conducted on the genotypes and molecular characterization of HBV in HIV-infected individuals in the Africa [39]–[42]. Such studies are important because the natural history of infection and response to antiviral therapy are influenced by the HBV genotype [40]. Thus the aim of this study was to molecularly characterize HBV isolated from HIV-infected southern Africans from the Mpumalanga Province cohort prior to the initiation of antiretroviral therapy (ART) [20], in order to determine the genotypes, possibly explain the high level of HBsAg- and HBeAg-negativity and to identify clinically relevant mutations.

## Methods

### Serum samples

Of the 298 samples obtained from HIV^+ve^ individuals prior to the initiation of antiretroviral therapy (ART), 71 plasma samples were shown to be positive for HBV DNA [20] and were used in this study. All participants from which the plasma samples were obtained signed informed consent. The study was approved by the Human Research Ethics Committee (Medical) of the University of the Witwatersrand and Mpumalanga Department of Health Research Ethics Committee. Although an attempt was made to sequence all 71 samples, the serological profile of those that were successfully sequenced is shown in Table 1.

**Table 1:** Characteristics of HBV isolated from HIV^+ve^ ART-naïve southern Africans.

Characteristics of Participant							Molecular characteristics of HBV isolates									Mutations^a^
							Basic core promoter/precore region								S region	
Serology	Sample	Sex	Age	ALT (IU/L)	CD4 (cells/ml)	HBVVL (IU/ml)	1762, 1764	1809–1812	1814–1816	1858	1862	1888	1896	Genotype	Subgenotype	
**HBsAg^+ve^ HBeAg^+ve^ anti-HBc^+ve^**	SHH121	M	33	22	125	1.02e+07	AG	TCAT	ATG	C	G	A	G	A	A1	-
	SHH159	F	23	38	105	1.33e+03	AG	TCAT	ATG	C	G	A	G	A	A1	ps1F25L, ps1V88L, ps2R48T
	SHH253	M	39	20	223	5.94e+02	AG	**TCCT**	ATG	C	G	A	G	A	A1	ps1V88A, ps2R48T, sE164V
	SHH255	F	36	17	34	1.82e+02	AG	TCAT	ATG	C	G	A	G	A	A1	ps1V88A, ps2R48T, sP120A, sE164V
	SHH274	F	52	39	182	1.10e+04	**TA**	TCAT	**ACG**	C	G	G	G	A	A1	***psDel***, ps1F25L, ps2Q10del, ps2R48T, ps2A53V, sP120T, sV168A
**HBsAg^+ve^ HBeAg^−ve^ anti-HBc^+ve^**	SHH001	M	23	38	172	3.59e+08	AG	TCAT	**CTG**	C	G	G	G	A	NS	NS
	SHH009	F	28	138	179	2.67e+05	**TA**	TCAT	ATG	C	G	A	G	A	NS	NS
	SHH011	F	25	21	156	1.39e+03	NS	NS	NS	NS	NS	NS	NS	NS	A1	***psDel***, ps1I48V
	SHH014	M	50	5	184	5.28e+03	NS	NS	NS	NS	NS	NS	NS	NS	A1	ps1F25L, ps1V88L, ps2Q10R, ps2R48T, ps2A53V, sQ129R, sV168A
	SHH016	M	34	55	9	6.25e+03	AG	GCAC	ATG	T	G	G	**A**	Non-A	NS	NS
	SHH022	F	19	28	132	3.35e+03	AG	TCAT	ATG	C	G	A	G	A	A1	-
	SHH048	F	29	7	255	1.58e+02	AG	TCAT	**TTG**	C	G	A	G	A	A1	-
	SHH070	F	41	290	246	5.16e+03	AG	TCAT	**CTG**	C	G	T	G	A	A1	-
	SHH100	M	27	86	148	6.31e+02	AG	TCAT	ATG	C	G	A	G	A	A1	**ps2M1L**
	SHH109	F	25	18	144	1.25e+07	AG	TCAT	ATG	C	G	A	G	A	A1	ps1V88A, ps2R48K
	SHH126	M	35	22	202	1.07e+07	AG	TCAT	ATG	C	G	A	G	A	NS	NS
	SHH148	M	47	32	99	1.49e+04	**TA**	**TCAC**	**AAG**	C	G	G	G	A	A1	-
	SHH167	M	38	129	49	2.86e+04	AG	TACT	ATG	T	G	A	**A**	Non-A	A1	***psDel***, ps1F25L, ps1V88L, ps2Q10del, ps2R48T, ps2A53V, sY100C
	SHH180	M	53	60	106	9.09e+04	**TA**	**TTCT**	ATG	C	G	G	G	A	NS	NS
	SHH240	M	35	35	27	2.93e+05	**AA**	**TCCT**	ATG	C	G	A	G	A	NS	NS
	SHH256	M	34	153	201	4.61e+02	AG	TCAT	ATG	C	G	A	G	A	NS	NS
	SHH300	M	33	24	98	3.30e+02	AG	**ACAT**	**ACG**	C	G	G	G	A	A1	***psDel***, ps1F25L, ps1V88L, ps2M1T, ps2Q10del, ps2R48T, ps2A53V
**HBsAg^+ve^ HBeAg^−ve^ anti-HBc^−ve^**	SHH042	F	30	11	289	1.98e+03	AG	GCAC	ATG	T	G	G	**A**	Non-A	NS	NS
	SHH055	F	24	8	261	4.00e+01	NS	NS	NS	NS	NS	NS	NS	NS	D3	-
**HBsAg^−ve^ HBeAg^−ve^ anti-HBc^+ve^**	SHH027	F	28	13	9	7.39e+02	AG	**TCAC**	ATG	T	G	G	**A**	?	NS	NS
	SHH029	F	27	28	245	1.14e+09	AG	TCAT	ATG	C	G	A	G	A	NS	NS
	SHH032	F	30	23	265	1.26e+03	AG	GCAC	ATG	C	G	G	**A**	A	A1	ps1I48T
	SHH039	M	38	30	46	2.79e+04	AG	TCAT	ATG	C	G	A	G	A	A1	ps1V88A, ps2R48T, sE164A, **sS174N** ¶
	SHH043	F	41	115	58	1.92e+03	NS	NS	NS	NS	NS	NS	NS	NS	A1	ps1I48V
	SHH044	F	64	35	103	1.43e+05	AG	TCAT	ATG	C	**T**	A	G	A	NS	**pcG1862T** ¶
	SHH045	F	18	8	151	6.33e+03	NS	NS	NS	NS	NS	NS	NS	NS	A1	***psDel***, ps1I48V, ps2M1I, ps2R48K
	SHH061	F	24	17	227	1.44e+03	**TA**	**TCTT**	ATG	C	G	T	G	A	NS	NS
	SHH074	M	47	4	68	9.47e+03	NS	NS	NS	NS	NS	NS	NS	NS	A1	ps1F25L, ps1V88L, ps2Q10R, ps2A53V
	SHH083	F	33	24	279	6.15e+05	AG	TCAT	ATG	C	G	A	G	A	NS	NS
	SHH094	F	34	24	196	7.17e+05	AG	**ACAC**	ATG	C	G	G	G	A	NS	NS
	SHH107	M	36	122	185	5.00e+01^b^	AG	TCAT	ATG	C	G	A	G	A	A1	sQ129R
	SHH110	F	25	20	148	1.01e+04	AG	TCAT	**CTG**	C	G	A	G	A	A1	sV168A, **sS174N** ¶
	SHH128	F	38	36	97	2.08e+03	AG	TCAT	ATG	C	G	A	G	A	NS	NS
	SHH130	M	61	36	47	6.57e+03	AG	TCAT	**CTG**	C	G	C	G	A	A1	ps1V88A, ps2M1I, sQ129H, sE164D, sV168A, **sS174N** ¶
	SHH131	F	28	7	255	1.12e+03	AG	TCAT	ATG	C	G	A	G	A	A1	-
	SHH132	M	45	17	44	4.98e+03	AG	TCAT	ATG	C	G	A	G	A	NS	NS
	SHH162	F	43	17	182	1.69e+03	AG	TCAT	ATG	C	G	G	G	A	NS	NS
	SHH193	F	23	25	12	4.39e+03	AG	**TCTT**	ATG	C	G	A	G	A	A1	ps1F25L, ps1V88L, ps2Q10R, ps2R48T, ps2A53V, sY100C, sQ129R, sV168A, **sS174N** ¶
	SHH217	F	23	16	173	1.01e+04	AG	GCAC	ATG	C	G	G	G	A	NS	NS
	SHH221	F	41	18	74	7.57e+03	**TA**	GCAC	**CTG**	C	**T**	A	G	A	A1	**pcG1862T** ¶, ps1I48V, sV168A
	SHH246	M	37	111	87	3.26e+04	AG	TCAT	ATG	C	**T**	A	G	A	NS	**pcG1862T** ¶
	SHH249	F	65	25	143	1.30e+04	AG	GCAC	ATG	C	G	G	G	A	NS	NS
	SHH264	M	28	24	101	9.02e+06	**TA**	TCAT	ATG	C	G	G	G	A	NS	NS
	SHH270	F	54	12	234	4.14e+03	NS	NS	NS	NS	NS	NS	NS	NS	A1	ps1F25L, ps1V88L, ps2Q10R, ps2R48T, ps2A53V
**HBsAg^−ve^ HBeAg^+ve^ anti-HBc^−ve^**	SHH187	M	63	14	87	9.38e+02	AG	TCAT	ATG	C	**T**	A	G	A	NS	**pcG1862T** ¶
**HBsAg^−ve^ HBeAg^−ve^ anti-HBc^−ve^**	SHH037	F	35	35	277	2.97e+06	NS	NS	NS	NS	NS	NS	NS	NS	A1	ps1I48V
	SHH053	F	37	45	196	8.60e+03	AG	GCAC	ATG	T	**T**	G	G	Non-A	NS	**pcG1862T** ¶
	SHH060	F	31	15	180	1.96e+02^b^	AG	GCAC	ATG	C	G	G	G	A	NS	NS
	SHH123	M	35	32	168	1.02e+05	AG	TCAT	ATG	C	G	A	G	A	NS	NS
	SHH173	M	34	8	110	5.86e+03	AG	TCAT	ATG	C	**T**	A	G	A	NS	**pcG1862T** ¶
	SHH184	F	51	5	225	6.45e+03	**TA**	**TCTT**	ATG	C	G	T	G	A	NS	-
	SHH219	F	39	4	214	1.98e+03	AG	TCAT	ATG	C	**T**	A	G	A	NS	**pcG1862T** ¶

a **:** -: no mutations, NS: no sequence, pc: precore, ps1:pre-S1, ps2:pre-S2, s:HBsAg,*psDel*: preS deletion,

¶ : occurred at significantly higher frequency in isolates from HBsAg^−ve^ individuals compared to HBsAg^+ve^ ones.

b **:** HBsAg^−ve^ individuals with HBV viral loads (HBVVL) <200 IU/ml representing true occult infection [21].

### Polymerase chain reaction (PCR)

DNA was extracted from 200 µl blood plasma with the QIAamp DNA Blood Mini Kit (QIAGEN Gmbh, Hilden, Germany) and eluted into 75 µl of best-quality water (BQW). Known positive and negative sera and BQW were used as controls for the extraction. The basic core promoter/precore (BCP/PC) region and complete S open reading frame (ORF) were amplified in a MyCycler™ thermocycler (Bio-Rad, Hercules, Ca, USA) using Promega Taq DNA polymerase (Promega, Madison, WI) as described previously in detail [20]. The BCP/PC region of HBV isolates was amplified using a nested PCR: primers 1606 (+) and 1974 (−) were used for the first round and 1653(+) and 1959(−) for the second round to yield an amplicon 1606–1974 from *EcoR*I site [41], [42]. A nested PCR reaction was carried out to amplify the complete S open reading frame: primers 2410(+)/1314(−)were used for the first round and 2451 (+) and 1280 (−)for the second round (2451–1260 from *EcoR*I site) [42].

### Sequencing

The amplicons were prepared for direct sequencing using the BigDye Terminator v3.0 Cycle Sequencing Ready Reaction Kit (Applied Biosystems., Foster City, USA) and sequencing was performed by the Central Analytical Facility, Stellenbosch University, South Africa, using the ABI 3130XL Genetic analyser (Applied Biosystems, Foster City, CA). BCP/PC sequences were analysed in both the forward and reverse directions of a single fragment, whilst the complete S sequences were analysed in 3 overlapping fragments. In addition to the primers used for amplification, HBV-specific primers [42] were used for sequencing.

### Phylogenetic analysis

Both BCP/PC (160 nt, 1742–1901 from *EcoR*I site) and complete surface DNA sequences (1203 nt, 2854–835from *EcoR*I site) were assembled and aligned manually using GeneDoc [43] and fed into MEGA5 [44]. The sequences were compared with corresponding subgenotype A1 sequences of HBV from GenBank. Nucleotide divergence calculations were carried out using Dambe [45]. The evolutionary history was inferred using the Neighbor-Joining method [46] and the evolutionary distances computed using the Kimura 2-parameter method [47]. Bootstrapping was performed using 1 000 replicates in order to determine the support for the specific nodes. The accession numbers of HBV isolates sequenced in this study have been deposited in GenBank/EMBL/DDBJ as JX144270–JX144323.

## Results

Using detection of HBV DNA by amplification of at least two of three regions of the HBVgenome, 71 of 298 HIV infected individuals were found to be co-infected with HBV [20]. The basal core promoter/precore (BCP/PC) region of 49 HBV isolates and the complete S region of 29 isolates were successfully sequenced. The relatively longer amplicon of the S region compared to the BCP/PC region, meant that fewer samples could be sequenced in that region successfully. The results are summarized in Table 1.

### Analysis of the basal core promoter/precore (BCP/PC) region

Using the criteria of Kramvis *et al* [26], the genotypes/subgenotypes were deduced from the BCP/PC region sequence of 48 isolates (Table 1). The genotype for SHH027 could not be deduced. Four isolates (SHH016, SHH042, SHH053, SHH167), with 1858T, did not belong to genotype A. Four isolates (SHH032, SHH060, SHH217, and SHH249) had Kozak sequence GCAC at 1809–1812, 1858C and 1888G, generally found in subgenotype A2. Thirty nine sequences belonged to subgenotype A1 with Kozak sequence TCAT (or mutant) at nucleotides1809–1812, 1888A and 1858C/T. Although SHH221, with 1858C belonged to genotype A, its subgenotype could not be deduced from the BCP/PC region because it had GCAC at 1809–1812 and 1888A.

Of the 49 cases, 5 were HBeAg^+ve^ and the remaining 44 HBeAg^−ve^. Three of the five isolates from HBeAg^+ve^ cases (SHH121, SHH159, SHH255) did not show any BCP/PC mutations, which can down-regulate or abolish the expression of the HBeAg. These had wild type sequences relative to the consensus for subgenotype A1 of HBV, that is, 1762A/1764G, 1809–1812 (TCAT Kozak), 1862G and 1888A. One isolate from a HBeAg^+ve^ participant, SHH253, had Kozak sequence TCCT, which would down- regulate but not abolish, HBeAg expression. Another isolate, SHH274, from a HBeAg^+ve^ participant, had a start codon mutation, together with 1762T/1764A. It is possible that HBeAg was expressed from a minor population.

The 44 BCP/PC sequences derived from HBeAg^−ve^ individuals were analysed for mutations (Table 1), which are known to down-regulate the synthesis of HBeAg or abolish its expression. In subgenotype A1, these mutations may be at the transcriptional, translational, or at post translational levels [48]. Mutations 1762T/1764A known to down-regulate HBeAg expression at the transcriptional level occurred in eight isolates (SHH009, SHH061, SHH148, SHH180, SHH184, SHH221 and SHH264, SHH274), in five cases occurring together with the T1753C. The Kozak mutations (1809–1812), affecting the expression of HBeAg at translational level were found in ten isolates, in two occurring together with the precore initiation codon mutations (1814–1816). Four isolates (SHH027, SHH094, SHH148 and SHH300) had either a single or double mutation or six isolates (SHH061, SHH180, SHH184, SHH193, SHH240 and SHH253) had a triple mutation at the Kozak sequence. Eight isolates had precore initiation codon mutations (table 1), which completely abolish HBeAg expression at translational level. The G1862T, which interferes with post-translational modification of the HBeAg-precursor and affects HBeAg expression [49] occurred in isolates from 7 HBeAg^−ve^ sera. The classical G1896A mutation occurred in five isolates and in four cases it occurred together with C1858T. An unusual mutation, T1884C, was detected in three HBV isolates (SHH0187, SHH219 and SHH246). Two of three isolates obtained from true occult infections were sequenced in this region: SHH060 and SHH107 and were wild-type for subgenotype A2 and A1, respectively (table 1).

Of the 39 samples belonging to subgenotype A1, for which the BCP/precore region was sequenced successfully, 18 were from HBsAg^+ve^ and 21from HBsAg^−ve^ sera. There was no difference in the frequency of the various BCP/PC mutations between the HBsAg^+ve^ and HBsAg^−ve^ groups, except for G1862T, which occurred in HBV from one third of HBsAg^−ve^ participants (7/21), but in none of the isolates from 18 HBsAg^+ve^ participants (p<0.05).

### Phylogenetic and molecular analysis of the complete S region

**Figure 1:**
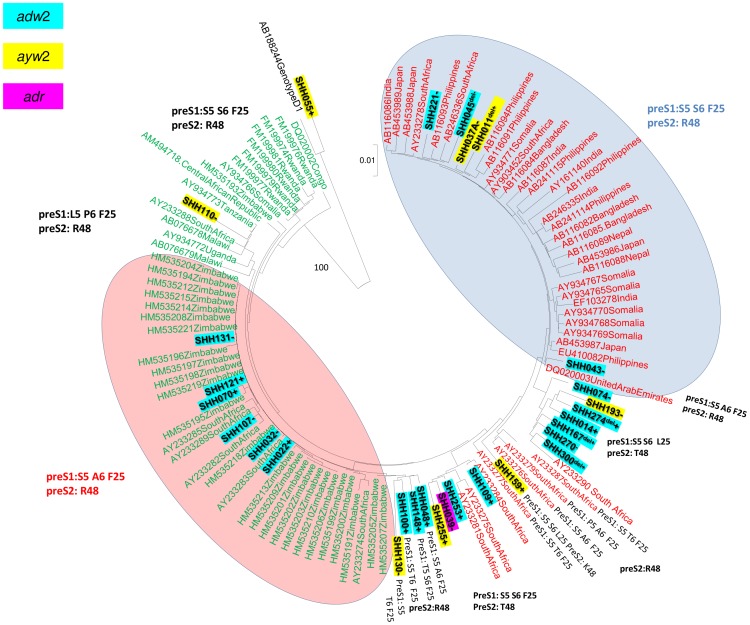
Phylogenetic relationship of complete pre-S1/pre-S2/S sequences (nt 2854–835 from the *EcoR*I site, numbering according to GenBank accession #AY233274) of 29 HBV isolates from HIV infected particpants [isolate number in bold, +: HBsAg^+ve^, −:HBsAg^−ve^, del: deletion mutant] to sequences of other African (*green*) and “Asian” (*red*) subgenotype A1 HBV isolates obtained from GenBank established using neighbour-joining. Bootstrap statistical analysis was performed using 1000 data sets and the numbers on the nodes indicate the percentage of occurrences. Each sequence obtained from GenBank is designated by its accession number and its country of origin. The characteristic amino acids in S open reading frame are indicated next to the sequences or relevant clades.

The complete S region (position 2854–835 from the *EcoR*I site) was sequenced successfully for HBV isolates from 29 participants. Following phylogenetic analysis, 28 of 29 isolates clustered with subgenotype A1, whereas one isolate, SHH055, clustered with genotype D (Figure 1) and belonged to subgenotype D3 (*data not shown*). For the isolate from SHH167, a discordant result was obtained between the HBV genotyping deduced using BCP/PC sequences and the S region phylogenetic analysis of HBV i.e. “not genotype A”and “subgenotype A1”, respectively.

Subgenotype A1 isolates from HBV/HIV co-infected individuals were compared to subgenotype A1 isolates from Asian and African countries using a circular unrooted phylogenetic tree (Figure 1). Seventeen isolates (SHH011, SHH014, SHH037, SHH039, SHH043, SHH045, SHH074, SHH109, SHH159, SHH167, SHH193, SHH221, SHH253, SHH255, SHH270, SHH274 and SHH300) clustered with the “Asian” cluster (*red*) and the remaining 11 isolates clustered with the African cluster (*green*). There was no clustering with respect to whether the isolates were derived from HBsAg^+ve^ or HBsAg^−ve^ samples.

Upon translation of the pre-S1/pre-S2, the majority of the subgenotype A1 isolates showed distinct subgenotype A1 amino acids Q54, V74, A86 and V91 in the pre-S1 region and L32 in the preS2 region [39], [50]. Twenty isolates belonged to serological subtype *adw*2, eight, including the genotype D isolate, to *ayw*2 and one to *adr* (Figure 1). The majority of the isolates in the “Asian” cluster (*red*) had S5, S6, F25 in the pre-S1. The isolates in the African cluster (*green*) displayed greater variation with three geographically distinct clades: the largest consisting of southern African strains with S5, A6, F25 in the pre-S1, a second consisting of eastern African strains with L5, P6, F25 and a third one consisting of central African strains, which like the “Asian” strains, had S5, S6, F25 in the pre-S1 (Figure 1). The majority of the isolates from the HIV-infected individuals sequenced in the present study displayed great variation at these signature positions (figure 1, table 1). The mean intragroup divergence ± standard deviation (%) of the complete S sequences of the newly sequenced strains from HIV-infected individuals was 2.43+0.12, whereas for the previously sequenced South African HBV subgenotype A1 isolates from HBV mono-infected individuals it was 1.92±0.75 [39].

**Figure 2:**
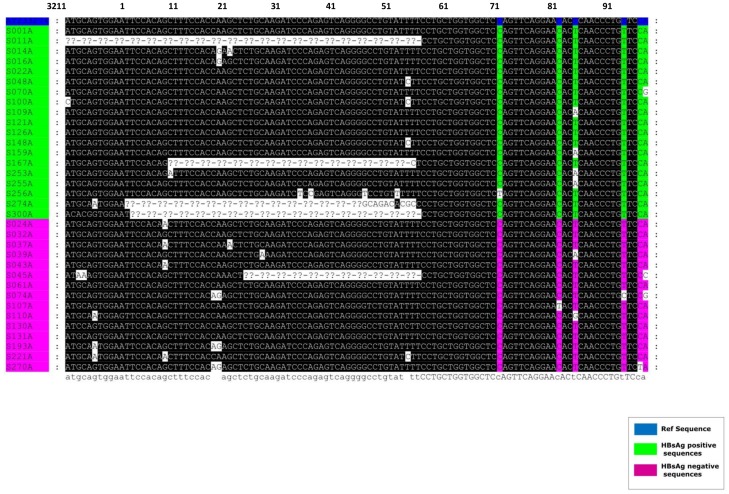
Deletions detected in the pre-S1/pre-S2 region of the HBV belonging to subgenotype A1 and isolated from HIV co-infected southern Africans. Position 1 corresponds to the *EcoR*I cleavage site of the HBV genome.

Five newly sequenced HBV isolates had deletions in the pre-S1/pre-S2 region (figure 2):

1. *SHH011 pre-S1 and pre-S2 deletion mutant:* This mutant strain had a double deletion. The first, a 30 nucleotide deletion found in the preS1 region at position 2900 to 2929 from the *EcoR*I site, leading to a 10 amino acid deletion of the pre-S1 amino acids 16–26. The second, a 66 nucleotide deletion in the pre-S2 region at nucleotide position 3211 to 55 from the *EcoR*I site, leading to a 22 amino acid deletion of the pre-S2 amino acids 1–22.
2. *SHH045 pre-S2 deletion mutant*: This mutant had a 33 nucleotide deletion at position 23 to 55 from the *EcoR*I site, leading to an 11 amino acid deletion of the pre-S2 amino acids 11–22.
3. *SHH167 pre-S2 deletion mutant*: This mutant had a 45 nucleotide deletion at position 9 to 54 from the *EcoR*I site, leading to a 15 amino acid deletion in the preS2 region.
4. *SHH274 and SHH300 pre-S2 deletion mutants:* These mutant strains had a 54 nucleotide deletion at position 2 to 55 from the *EcoR*I site, leading to an 18 amino acid deletion in the pre-S2 region.

All deletion mutants, except for SHH045, were from HBsAg^+ve^ participants.

**Figure 3:**
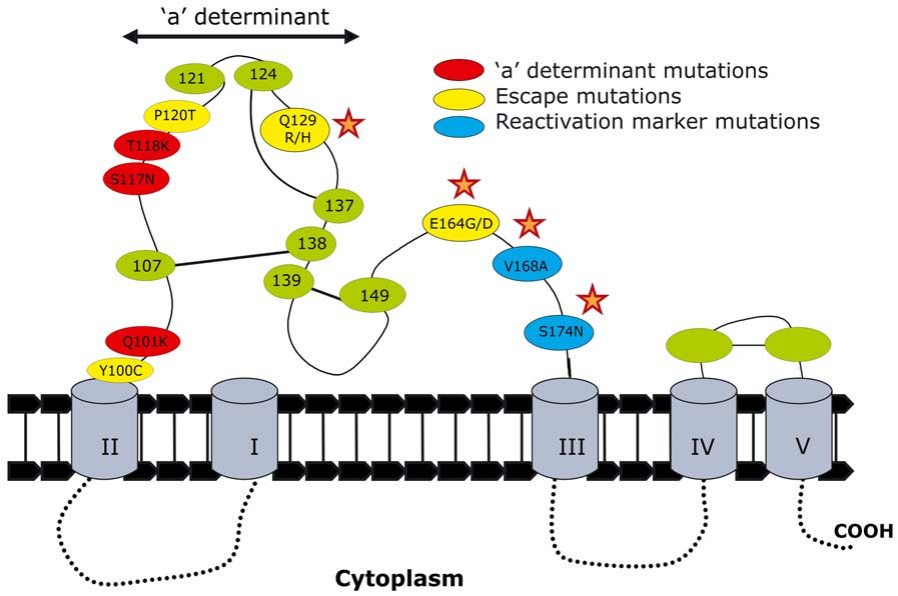
Graphic representation of mutations found within the small envelope protein of the HBV isolated from HIV infected participants. This is a hypothetical representation [91]. The mutations marked with a star occurred significantly more frequently in HBV isolated from HIV-co-infected individuals in this study than in strains of the same cluster of the phylogenetic tree (figure 1).

The following mutations occurred significantly more frequently in HBV isolated from HIV-co-infected individuals in this study than in strains of the same cluster of the phylogenetic tree: in the pre-S1, ps1F25L, ps1V88L/A; in the pre-S2, ps2Q10R, ps2 R48K/T, ps2A53V and in the S region sQ129R/H, sQ164A/V/G/D, sV168A and sS174N (p<0.05). In the pre-S1, ps1I48V/T occurred more frequently in females than males (p<0.05). Isolates with sV168A occurred more frequently in participants with viral loads greater than 200 IU per ml (p<0.05). A mutation in ps2M1 of the preS2 abolished the start codon in four isolates. When comparing the frequency of mutations in HBsAg^+ve^ and HBsAg^−ve^ individuals, only sS174N occurred more frequently in HBsAg^−ve^ individuals (p<0.05). sQ129R was the only mutation detected in the only isolate sequenced from a true occult HBV infection (SHH107, viral load <200 IU/ml) [21]. The relevant S region mutations are shown relative to the ‘a’ determinant (Figure 3).

### Molecular analysis of the polymerase region

In the polymerase region, the following mutations occurred significantly more frequently in HBV isolated from HIV-co-infected individuals than in isolates of the same cluster on the phylogenetic tree: spQ23K, spL28P, spS91I, spP132Q, spQ125E and rtE1D (p<0.05). The unusual start codon mutation, rtE1D, was seen in eight isolates, which grouped in the “Asian” cluster following phylogenetic analysis. This mutation occurred together with rtS105T+rtH122N in 7 isolates and with rtQ125E in three isolates. Analysis of 457 sequences from GenBank revealed that the rtE1D mutation was found in genotype B and G isolates from Asian countries. Glutamic acid (E) and aspartic acid (D) have similar chemical structures and properties; therefore this mutation is not expected to introduce a significant functional change to the reverse transcriptase polymerase and the isolates are probably replicative. Three isolates had drug resistant mutations: SHH011 had rtV214A, SHH074 had rtL180M+rtM204V and SHH130 had rtV173L. There was no significant difference in the frequency of polymerase mutations in HBV from HBsAg^+ve^ and HBsAg^−ve^ individuals. Only one of three isolates (SHH107) obtained from true occult infections was sequenced in this region and found to contain mutations only in the spacer region of the polymerase.

## Discussion

Compared to areas of low endemicity, where HIV and HBV are most likely transmitted at the same time during sexual maturity, in southern Africa, where both viruses are endemic, HBV infection occurs before the age of 5 years and these children become chronic carriers of HBV in adulthood [8]. Therefore, the majority of South Africans are naturally protected by antibodies to HBV by the time they acquire HIV at the age of sexual maturity [5]. In a recently completed study of 298 participants, 231 (77.5%) showed at least one HBV marker, 134 (45%) were anti-HBs^+ve^, either alone (11; 3.7%) or together with anti-HBc (123; 41.3%) [20]. However, immunosuppression, as a result of HIV infection, can lead to HBV infection and/or reactivation [51]–[53], increasing the frequency of HBV infection in HIV^+ve^ participants with previously resolved HBV infection. Of the 231 participants exposed to HBV, 53.7% were HBV DNA^−ve^ (resolved) and 23.8% HBV DNA^+ve^ (current) [8.7% HBsAg^+ve^: 15.1% HBsAg^−ve^] [20]. In the present study we determined the HBVgenotypes and molecularly characterized the HBV isolated from these HBV and HIV- co-infected southern Africans.

Subgenotype A1 was found to be the most prevalent subgenotype in rural South African HIV-infected individuals. In agreement with others, who found a predominance of genotype A of HBV in HIV infected individuals [54], [55], we found that the ratio of genotype A to non-A (97% to 3%) was higher in the HBV/HIV co-infected individuals compared to mono-infected individuals. Previous genotyping showed a 75%∶25% ratio of genotype A to D in South African mono-infected asymptomatic carriers and liver disease participants [39] and HBsAg^−ve^ blood donors [56], whereas this was higher in HBsAg^+ve^ blood donors (90%∶10%). Discordant results between the genotypes deduced when the BCP/PC region and when the S region was sequenced were obtained for isolate SHH0167 (table 1). It is possible that this participant was infected with both genotypes A and D or with a genotype A/D recombinant, which has been shown to occur in South Africa [40]. The only way that this could be differentiated would be by carrying out full genome amplification to identify the recombinant and/or cloning to identify the mixed population.

Upon phylogenetic analysis, the HBV isolates were found in both African and “Asian” clusters (Figure 1), intimating a high diversity in the strains circulating in the rural cohort residing in a relatively small geographical region. This high diversity may be indicative of the high mobility of populations to and from this region. The cohort site, in the Shongwe region, is close to the borders with Swaziland and Mozambique. High levels of migration from these surrounding countries [57], may lead to higher risk of sexually transmitted infections including HBV and further increased genetic variability of HBV. A number of mutations occurred significantly more frequently in HBV isolated from HIV-co-infected individuals in this study than in strains of the same cluster of the phylogenetic tree (figure 1). This was probably as a result of the immunosuppression, which can alter the evolutionary rate of the virus [58]. The isolates from the African cluster showed greater variation than the “Asian” cluster, which comprised mostly Asian subgenotype A1 isolates, with some South African and Somalian isolates. This concurs with the hypothesis that subgenotype A1 has been endemic in the African population for a long period of time [1].

The HBeAg negativity found in 44/49 Shongwe participants (89,7%) could be accounted for by the following HBV mutations: the basic core promoter mutations A1762T/G1764A, which can down-regulate transcription of precore mRNA [59]; the Kozak sequence mutants that affect HBeAg translation [60]; precore start codon mutations that abolish HBeAg expression [61], the G1862T mutation,which interferes with post-translational modification of the HBeAg-precursor [49], [62], and the classical G1896A stop codon mutation with C1858T [63]. The 1762T/1764A mutations have been closely related to progression of chronic liver disease [41], [64], [65] and together with T1753C, found in five Shongwe HBV isolates, have been described as markers for HCC [66]–[68]. The G1862T mutation occurred in HBV from HBsAg^−ve^ participants but not in HBV from HBsAg^+ve^ participants (p<0.05). This mutation causes intracellular retention of the HBeAg precursor, aggresome formation and impaired secretion of HBeAg [49]. It is possible that the intracellular retention of the HBeAg interferes with the expression of HBsAg, leading to HBsAg-negativity.

Pre-S/S sequence data were analyzed in an attempt to explain the high HBsAg-negativity, which was a feature of this rural cohort of HBV/HIVco-infected individuals [20]. Five HBV strains isolated from HBV/HIV co-infected participants had pre-S deletions. Only one of the five participants from which these strains were isolated was HBsAg^−ve^. All five had pre-S2 deletions, ranging in size from 11 to 22 amino acids, with one isolate having, in addition, a 10 amino acid pre-S1 deletion. Deletion mutants in the pre-S region have been previously found to occur more frequently in HIV-coinfected individuals [69] and in HCC patients [70]. Deletion mutants in the pre-S region have been reported to cause overproduction and accumulation of LHBs protein in the endoplasmic reticulum (ER), which causes significant ER stress that may induce DNA damage and genomic instability and hence play a possible role in hepatocarcinogenesis [71], [72]. The pre-S mutants may contribute to viral oncogenesis by transcriptional activation of the viral promoter elements [73]. A higher oncogenic potential of pre-S2 deletions has been found compared with that of pre-S1 deletion mutants [74], with pre-S1 deletion mutants displaying different phenotypes to pre-S2 deletion mutants, when transfected in Huh-7 cells [75].

Although, with the exception of sS174N, there was no significant difference between the frequency of the mutations in the S region from HBsAg^+ve^ and HBsAg^−ve^ participants, there were a number of mutations that could account for the inability to detect HBsAg. These included ps1F25L, ps1I48V, ps1V88L/A in the pre-S1 region, ps2M1I, ps2Q10R, ps2R48K/T, ps2A53V in the pre-S2 and sY100C, sP120T/A, sQ129R/H, sE164D and sS174N in the S region (figure 3). Pre-S1 residues 21–96 contain the virus neutralising epitope and a hepatocellular binding site, and the preS2 residues 1–11 and 21–47 have been shown to mediate HBV attachment to hepatocytes [76]. Therefore mutations in these regions may lead to conformational changes in the LHBs and MHBs, which may result in HBsAg negativity. ps2Q10R is in the major pre-S2 antigenic region known to carry numerous B cell, T-helper cell and cytotoxic T-lymphocyte epitopes [77] and may therefore reduce the binding ability of the antibodies to the epitope and interfere with their neutralising effect. sY100C has previously been detected in subgenotype A1 isolated from occult hepatitis participants [70], [71] and has been associated with HBsAg-negativity in blood donors [78]. sP120T, which also leads to an rt128N mutation in the polymerase, has been detected in participants with severe hepatitis following lamivudine (LAM) and HBIg treatment [79] and can partially restore the replicative capacity of LAM-resistant HBV *in vitro* [80]. sP120T and sQ129R/H fall within the ‘a’ determinant and would therefore affect its antigenic ability and infectivity [81]. sQ164A, has been shown to reduce the antigenicity of HDV particles [81] and sE164D, with the concomitant rtV173L, a lamivudine escape mutant [82], has reduced affinity for anti-HBs antibodies *in vitro*, similar to that of the classical G145R [83].

Of interest was the presence of previously identified reactivation markers [84]. The reactivation markers V168A occurred together with S174N, which was only found in isolates from HBsAg^−ve^ but not HBsAg^+ve^ participants. HBV with V168A occurred more frequently in participants with higher viral loads. These have previously been detected in a serologically-negative HBV/HIV co-infected patient following a symptomatic HBV reactivation [84].

The S region mutations detected in the present study differed from those detected previously in HBsAg^−ve^ blood donors, with true occult HBV infection and low viral loads of subgenotype A1 [56]. The HBV mutants in the present study had viral loads >200 IU/ml, escaped detection by HBsAg assays and probably arose during reactivation or were transmitted to these unvaccinated individuals, in the absence of an immune response or exogenous selective pressure such as vaccination or ART. The presence of minority populations in the quasispecies expressing wild-type HBsAg may account for the fact that these mutations were also isolated from HBsAg^+ve^ individuals. This possibility is being investigated using ultra-deep pyrosequencing.

Even though the participants in the present study had not initiated ART, ten percent, 3 of 29 isolates sequenced, had drug resistance mutations rtV173L, rtL180M+rtM204V and rtV214A, respectively. Mutants rt173L and rt180M have been shown to restore viral replication in the presence of LAM, whereas rt204V results in reduced replication in *in vitro* transfection studies [85], [86]. In South Africa, rtM204I has been detected in therapy-naïve HBV/HIV co-infected individuals [87] and rtM204V in treated HBV mono-infected participants [88]. All the mutations described occurred in genotype A. Compared to other genotypes, genotype A in HBV-HIV co-infected participants has been shown to be more prone to immune/vaccine escape mutants, pre-S mutants associated with immune suppression, drug associated mutations and HCC [69], [89], [90].

In conclusion, the study showed that subgenotype A1 predominates in HBV/HIV co-infected individuals from rural South Africa. Subgenotype A1 HBV isolates had mutations that can affect HBeAg-expression at the transcriptional, translational and posttranslational levels and these mutations can account for the HBeAg negativity seen in the majority of HBV/HIV infected individuals in this cohort. Although there were no significant differences between all S region mutations occurring in HBV from HBsAg^+ve^ and HBsAg^−ve^ individuals, pre-S region mutations and deletions, and ‘a’determinant or immune/vaccine escape mutants may account for HBsAg negativity seen in some participants. Moreover, these mutants may escape detection by standard commercial serological tests currently used in South African health services. HBV infection will remain undetected unless nucleic acid testing, which can detect HBV DNA in the presence or absence of HBsAg in the serum, is implemented. Deletion mutants, previously shown to occur in HBV from HCC patients, were detected in the present study. These could be a risk factor for the development of HCC in HIV patients, whose lifespan is being increased and immune system reconstituted following the introduction of ART. Thus more studies are necessary to functionally characterize these deletion mutants. Furthermore, the presence of mutants resistant to LAM, prior to the initiation of LAM-containing ART, has important implications and repercussions, and highlights the need for the inclusion in the treatment regimen of tenofovir (TDF), to which these mutants are sensitive. This study has provided important information on the molecular characteristics of HBV in HIV-infected South Africans prior to the initiation of ART. Our findings have important clinical relevance in the management of HBV and HIV co-infection in our unique setting, where subgenotype A1 HBV is hyperendemic and usually transmitted horizontally in childhood, before HIV infection occurs in adulthood.
